# Optimization of Cu/Sn Alloy Sputtering Process Based on Orthogonal Experimental Design Method

**DOI:** 10.3390/mi14081539

**Published:** 2023-07-31

**Authors:** Shuangjie Liu, Xingwang Li, Yongping Hao, Xing Li, Fengli Liu

**Affiliations:** 1 School of Equipment Engineering, Shenyang Ligong University, Shenyang 110159, China; shuangjieliu@126.com (S.L.); yphsit@126.com (Y.H.); 13624057067@163.com (X.L.); 2 School of Mechanical Engineering, Shenyang Ligong University, Shenyang 110159, China

**Keywords:** magnetron sputtering, copper–tin alloy film, electrical conductivity, orthogonal experimental design, uniformity

## Abstract

The performance of supercapacitors is directly influenced by the conductivity of polypyrrole, which serves as the electrode material. In order to balance considerations of cost-effectiveness and conductivity, this study employs magnetron sputtering to fabricate a copper–tin alloy layer as the conductive layer for polypyrrole. The deposition of a copper–tin alloy film through magnetron sputtering has a significant impact on the polymerization effect of pyrrole as well as being a crucial factor influencing the performance of supercapacitors. Various parameters, including working pressure, sputtering time, and sputtering power, affect the conductivity of the copper–tin alloy film. Furthermore, the degree of influence of each parameter on the conductivity of the copper–tin alloy film varies. This study utilizes an orthogonal experimental design to investigate the impact of various factors and levels on the conductivity and uniformity of a metal film. The objective is to optimize the process parameters for the creation of a copper–tin alloy film with desirable characteristics. Experimental results indicate that the working voltage, sputtering time, and sputtering power significantly influence the coefficient of variation, deposition rate, target current, and operating voltage of the film. Furthermore, FT-IR, XRD, and SEM tests are conducted on samples prepared using the identified optimal process parameters. In addition, we demonstrate various approaches to enhance the experiment’s reliability. The findings indicate that the most favorable process parameters for achieving optimal results are a working pressure of 0.065 Pa, a sputtering time of 20 min, and a sputtering power of 70 W. It was observed that the sputtering time significantly influences the uniformity of the copper–tin alloy film, whereas the sputtering power has a minimal impact on its uniformity. The deposition rate is primarily influenced by the working pressure, with the greatest effect observed. Conversely, the sputtering time has the least impact on the deposition rate. Similarly, the target current is predominantly affected by the sputtering power, exhibiting the greatest influence, while the sputtering time has the least effect. Furthermore, the working voltage is most significantly influenced by the working pressure, whereas the sputtering time has the least impact on the working voltage.

## 1. Introduction

The supercapacitor, characterized by its high specific capacitance, high power density, wide temperature range, compact size, affordability, ease of material acquisition, and prolonged lifespan [1], has emerged as a novel and environmentally friendly energy storage device. Consequently, extensive research has been conducted to explore its potential as a new energy storage component in various domains, owing to the advancements and establishment of supercapacitors.

The fundamental configuration of a supercapacitor comprises two plates featuring electrodes and a dielectric layer. When charging, the charge is accumulated on the electrode plate, resulting in an electrode–electrolytic dielectric–electrode arrangement. The selection of electrode material significantly impacts the maximum voltage, capacitance, and stability. Among the numerous electrode materials available, conductive polypyrrole (PPy) exhibits favorable attributes such as flexibility, redox properties, and exceptional stability, rendering it highly favored by researchers as an electrode material for supercapacitors [2]. Currently, the predominant techniques for synthesizing conductive polypyrrole (PPy) are chemical oxidation and electrochemical polymerization. The chemical oxidation method enables the simultaneous production of a substantial quantity of conductive polymers as it involves an oxidation-coupling process. This method is straightforward and allows for the synthesis of conductive polymers over a large area. Additionally, under suitable conditions, soluble conductive polymers can be synthesized. However, the resulting polymers exhibit numerous flaws due to the limited selectivity of the oxidant, and they are obtained in a powdered form. The electrochemical polymerization method involves the sputtering of a PVDF film with a metal layer on both sides, serving as a working electrode. This allows for the direct occurrence of an oxidative polymerization reaction on the film’s surface, resulting in the deposition of a conductive polypyrrole film. In contrast to the chemical oxidation method, the electrochemical polymerization method offers the ability to alter the surface morphology and properties of the conductive polypyrrole by manipulating the doping ion type and polymerization reaction parameters. Hence, electrochemical polymerization is the preferred technique for the synthesis of conductive polypyrrole. The crucial aspect of this method lies in the quality of metal film sputtering. Any non-uniformity in the metal film, such as surface roughness or granular protrusions, can significantly impact the conductivity of the film. Subsequently, during the subsequent step of electrochemical polymerization, the uneven polymerization of pyrrole and varying thickness may impede the formation of polypyrrole, thereby compromising the performance of supercapacitors.

The orthogonal test method is a design methodology employed to investigate multi-factor and multi-level scenarios. It involves selecting representative horizontal combinations from a comprehensive test for evaluation purposes, which is followed by analyzing the results to identify the optimal level combination. In the literature [3], Wang Bo conducted a study on the impact of three influential factors, namely substrate temperature, sputtering air pressure, and sputtering power, on the adhesion of Mo film. This investigation utilized orthogonal experiments to optimize the parameters of the preparation process. In the literature [4], the focus is on addressing the issue of the coexistence of various vanadium oxides when using traditional methods to prepare VO_2_ film components. To overcome this problem, an orthogonal test is conducted to prepare VO_2_ film using RF magnetron sputtering on a sapphire substrate with a VO_2_ target. Subsequently, annealed heat treatment is applied, and the laser protection of the resulting film is significantly improved through parameter optimization. The evaluation index in the literature [5] is the nanohardness and bonding strength of the film. The study analyzes the influence and mechanism of four process parameters (sputtering target power, substrate temperature, argon flow, and vacuum degree) on the comprehensive mechanical properties and microstructure of the sputtering MO film. Additionally, the study [6,7] examines the impact of three key factors (sputtering power, pressure, and substrate speed) on the uniformity, compactness, and adhesion of the aluminum film and optimizes the obtained results. The study investigated the impact and interplay of magnetron sputtering process parameters on the surface roughness of Ti_6_Al_4_V alloy through an analysis of the literature [8]. Regression analysis and variance analysis were employed to identify the optimal process parameters and evaluate the model’s adequacy. The research also examined the influence of sputtering parameters on the surface roughness of coating samples and made predictions regarding surface roughness. In [9], the authors investigate the impact of power, deposition time, temperature, and working pressure on the resistivity, deposition rate, and sensitivity of titanium dioxide thin film through the utilization of RF magnetron sputtering technology. Additionally, the optimal process parameters are determined. In a separate study, the authors in [10] explore the deposition of cerium dioxide (GDC) doped film using the magnetron sputtering method while examining the influence of process parameters on the properties of the film. The optimization of the preparation process for hafnium oxide nitride (HfOxNy) film is conducted [11], resulting in the attainment of the material with the most favorable electrical parameters. During the optimization process, the dielectric film’s parameters are monitored through electrical characterization of the MIS structure, utilizing hafnium oxide nitride as the gate-dielectric. Additionally, the thermal stability of the prepared HfOxNy layer is investigated. The impact of various process parameters, including oxygen pressure, substrate temperature, and annealing treatment, on the structural, magnetic, and transport characteristics of thin films, as well as their stoichiometry, has been explored in the literature [12,13,14]. Additionally, ref. [15] investigated the influence of sputtering pressure on the microstructure, morphology, and electrochromic properties of tungsten trioxide films, with a focus on optimizing the process parameters.

In the literature reviewed, the orthogonal test method plays an important role in experimental design and analysis. It is able to identify critical factors, optimize experimental protocols, and reduce uncertainties, and it can save resources. By systematically considering multiple factors, the orthogonal test method provides an efficient and reliable method to study and optimize experiments. Specifically, the working pressure, sputtering time, sputtering power, and argon flow rate employed in the sputtering process investigated in this study exert an influence on the conductivity of the copper–tin alloy film. Furthermore, the extent of influence varies for each factor affecting the conductivity of the copper–tin alloy film. The performance of supercapacitors and their engineering applications are largely dependent on the quality of copper–tin alloy film sputtering. Hence, orthogonal tests are devised to identify the optimal level match with a reduced number of tests. By optimizing the process parameters, a copper–tin alloy film with desirable conductivity and uniformity can be fabricated, which holds significant importance for the practical implementation of copper–tin alloy film in engineering applications. In the context of supercapacitors, the unhindered migration of ions between the positive and negative electrodes is crucial for effective charge storage and release. Nevertheless, the excessive thickness of the copper–tin alloy film impedes ion migration, thereby restricting the charge transfer between the electrodes and ultimately diminishing the supercapacitor’s performance. An insufficient thickness and low sputtering quality of the magnetron-deposited copper–tin alloy film can lead to undesired pyrrole polymerization or hinder the formation of polypyrrole. Consequently, the preparation of a uniform and smooth metal layer using the orthogonal test method becomes imperative for the production of Cu–Tn alloy films intended for engineering applications. Therefore, this study employed a DC magnetron sputtering technique to deposit a thin copper–tin alloy onto the PVDF matrix while simultaneously optimizing the parameters of the preparation process.

## 2. Experiment

### 2.1. Experimental Principle

**Figure 1:**
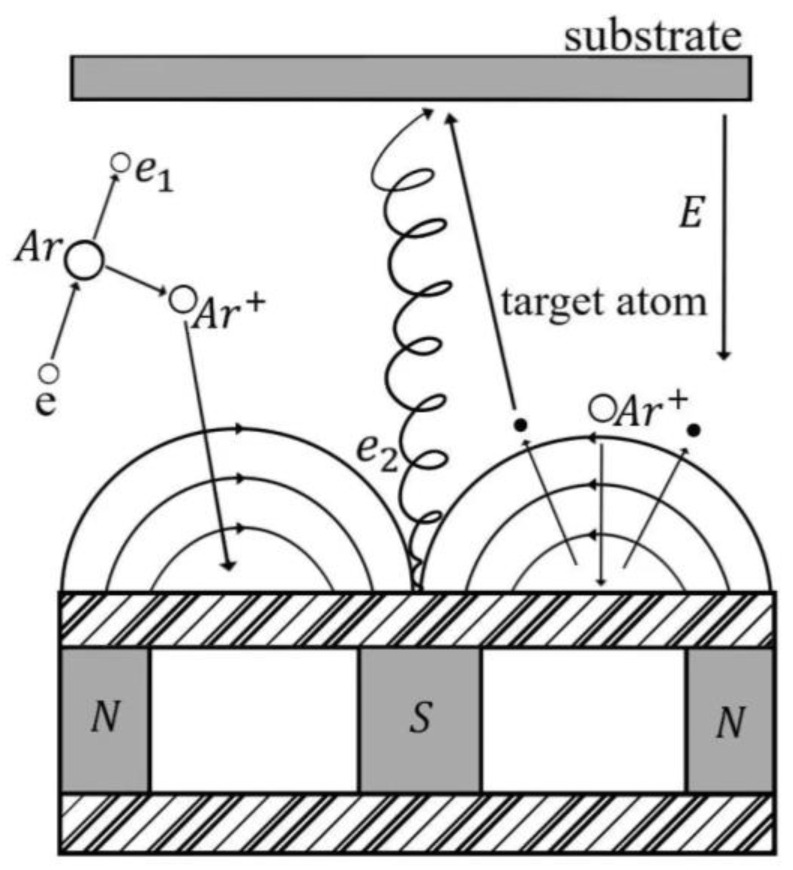
Schematic diagram of magnetron sputtering.

The JZCK-450-2A magnetron sputtering coater, sourced from Shenyang Juzhi Vacuum Equipment Co. (Shenyang, China), was employed as the experimental instrument. Figure 1 presents the schematic diagram illustrating the principle of magnetron sputtering.

### 2.2. Experimental Process

The experimental study employed a copper–tin alloy target characterized by a cop-per–tin quality ratio of 8:2 and a purity level of 99.99%. The selection of a copper–tin alloy (mass ratio 8:2) as the target material is primarily motivated by several key factors. Copper, due to its remarkable electrical conductivity and cost-effectiveness, has been chosen as the primary constituent. Furthermore, copper exhibits a consistent electrical conductivity across varying temperatures, rendering it suitable for deployment in diverse environments and operational circumstances, thereby underscoring its significance in engineering applications. The incorporation of tin into the alloy enhances its hardness and strength, rendering it more suitable for withstanding elevated forces and pressures in particular applications. Moreover, copper–tin alloys exhibit notable resistance to corrosion and demonstrate greater resilience against oxidation, corrosion, and diverse corrosive agents compared to pure copper. Consequently, copper–tin alloys possess enhanced versatility relative to pure copper. The substrate utilized was micron PVDF, featuring a pore size of 0.45. Prior to the commencement of the experiment, the target material underwent pretreatment, involving the grinding and polishing of the copper–tin alloy using sandpaper of varying specifications [16,17]. Subsequently, impurities were eliminated through the application of absolute ethanol, which was followed by ultrasonic cleaning to achieve a target surface of optimal smoothness. The desired distance between the target base is 7 cm, the sputtering atmosphere consists of high-purity argon, the vacuum level in the working chamber should be reduced to below 2 × 10^−3^ Pa before commencing the deposition process, and the sputtering temperature is maintained at room temperature. Prior to the formal coating, the target undergoes a pre-sputtering phase lasting 5 min, which serves the purpose of eliminating any extraneous materials from the target surface, thereby guaranteeing the purity of the target atoms that are subsequently sputtered onto the substrate.

### 2.3. Orthogonal Experimental Design

This paper utilizes the L_9_(3^4^) orthogonal table for testing purposes, with three factors (A, B, C) representing working pressure, sputtering time, and sputtering power, respectively. D indicates an empty column, as shown in Table 1. The selection of working pressure, sputtering time, and sputtering power was informed by the existing literature and prior experimental experience. The evaluation index for the orthogonal test was the conductivity of the copper–tin alloy film. The sample, prepared through magnetron sputtering, was measured using the YT1004 electronic analytical balance (Ucovit Electronic Technology Co., Ltd., Kunshan, China)under various preparation parameters. The uniformity and conductivity tests were conducted using the KEITHLEY 2636B source meter(Keithley Instruments, Inc., Ohio, USA). The assessment of the average voltage drop in the conductivity test was used to determine the conductivity of the copper–tin alloy film. The measured sample is cut into a 2.5 cm × 2.5 cm square, the current of 5 mA is output by using the KEITHLEY 2636B source meter, the voltage drop at the center of each side is measured from the center of the sample, and each measurement from the center of the sample to the center of each side (up, bottom, left, right) is recorded as A, B, C and D in turn. Then, the average voltage drop is calculated. The higher the conductivity of the test sample, the lower the average voltage drop.

For each factor, three different levels (r = 3) were selected, and each level was repeated three times (m = 3) in the protocol, resulting in a total of nine sets of tests (*n* = 9) that needed to be completed. In addition, the selection of the range for each factor level was based on existing literature and previous experimental experience.

In the context of orthogonal experimental design, it is important to acknowledge that the experimental equipment employed may introduce a degree of error in the obtained results [18]. This error typically arises from the inherent limitations of the experimental equipment, the precision of the measuring instrument, and the proficiency of the operator. The presence of experimental errors can potentially impact the outcomes of orthogonal tests by augmenting the variability of the collected data and diminishing the overall accuracy and reliability of the experiment. Consequently, this may lead to an inaccurate assessment of certain test factors as well as the potential masking or amplification of specific effects. This study employs strategies to mitigate the impact of experimental equipment errors on orthogonal tests.

In the first instance, factor D represents the empty column, which assumes a crucial role in statistical analysis. An empty column is a means to identify and estimate error terms or unaccounted factors in the experimental outcomes. Typically, it is necessary to include at least one empty column. If there are no empty columns, then the experiment should be repeated with each set of levels. The inclusion of an empty column serves as an indicator, to a certain degree, of the potential influence of the experimental equipment on the test results. In addition, prior to each magnetron sputtering, the experimental order was randomized rather than adhering to the order specified in Table 2. Randomly selecting the experimental order serves to equalize the influence of the experimental equipment on the outcomes, mitigate the sequence effect, and validate the reliability of the findings. The random selection of the experimental order effectively diminishes the impact of experimental errors on the results, thereby enhancing the reliability and comparability of the experimental outcomes. This serves to augment the scientific rigor of the study and guarantees the precision and credibility of the experimental findings. It is worth noting that despite the anticipation of unfavorable results in certain trials based on relevant expertise, it is crucial to carry out all the trials. This is because each set of trials yields valuable information from various perspectives. Finally, in each experiment, other confounding factors were controlled to minimize their effect on the results.

## 3. Results

### 3.1. Analysis of Orthogonal Test Results

**Figure 2:**
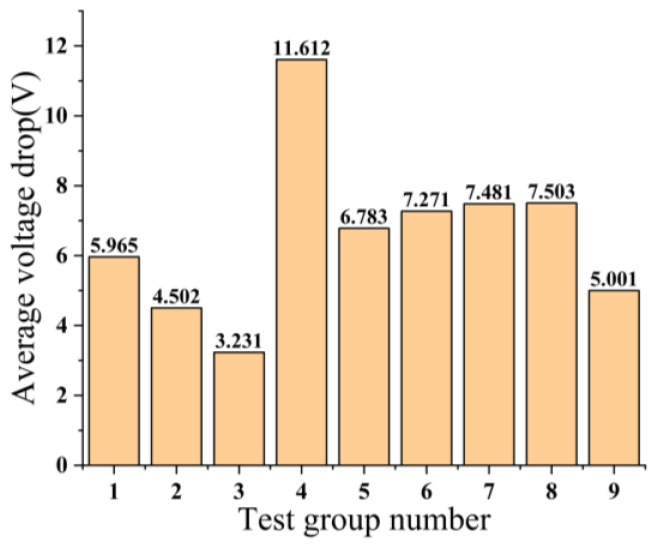
Results of 9 sets of experiments.

The chosen evaluation index for this orthogonal test is the conductivity of the copper–tin alloy film, specifically referred to as the average voltage drop. A lower average voltage drop indicates a higher conductivity of the copper–tin alloy film. A total of nine sets of tests are required to be conducted, with the results of each test denoted as y_1_, y_2_, … y_9_. These test outcomes are illustrated in Figure 2.

According to the data obtained from the experiment, the observed values of each statistic are calculated separately, and the calculation results are shown in Table 3. For column *j* (including empty columns) in the orthogonal table, $K_{jl}$ is the sum of the three test results of horizontal l (l = 1, 2, 3) in column *j*.

(1) $$K=\sum_{l=1}^{r}K_{jl}=\sum_{i=1}^{n}y_{i}$$

From Equation (1), *K* is the sum of all test results, so *K* has nothing to do with *j*. Order:

(2) $$P=\frac{1}{n}K^{2}$$

(3) $$Q_{j}=\frac{1}{m}\sum_{l=1}^{r}K_{jl}^{2}$$

(4) $$S_{j}^{2}=Q_{j}-P$$

(5) $$Q=\sum_{i=1}^{n}y_{i}^{2}$$

(6) $$S_{T}^{2}=Q-P$$

(7) $$\bar{y}=\frac{1}{n}K$$

And from Equation (7), we know

(8) $$S_{T}^{2}=\sum_{i=1}^{n}{\left(y_{i}-\bar{y}\right)}^{2}=\sum_{j}S_{j}^{2}$$

The significance of the corresponding factor is represented by the magnitude of the sum of squared deviations $S_{j}^{2}$ in Table 3. A larger $S_{j}^{2}$ indicates a greater impact of the factor on the evaluation index. The $S_{j}^{2}$ values for the three factors, namely working pressure, sputtering time, and sputtering power, are observed to be 23.893, 15.711, and 2.641, respectively. The $S_{j}^{2}$ value of the empty column is 3.662. Upon calculation, it is determined that the respective percentage contributions of working pressure (Factor A), sputtering time (Factor B), sputtering power (Factor C), and null column (Factor D) are 52.047%, 34.224%, 5.753%, and 7.976%. Furthermore, the combined percentage contribution of working pressure, sputtering time, and sputtering power amounts to 92.024%, signifying that factors A, B, and C collectively account for a significant portion of the impact on the electrical conductivity of the metal film. This phenomenon holds significant importance despite the higher percentage contribution of the null column (factor D) compared to factor C. Due to the multitude of factors influencing the presence of empty columns, the relative contribution of a particular factor is significantly less pronounced compared to the contribution of factor C. Consequently, the outcomes of this experiment indicate that the primary influencing factor is the working pressure (factor A), followed by the sputtering time (factor B), while the sputtering power (factor C) exhibits the least influence. Based on the findings derived from conducting nine sets of tests, it is evident that the third group of tests exhibited the lowest average voltage drop, thereby indicating that the copper–tin alloy film prepared under the conditions of A_1_B_3_C_3_ demonstrated superior conductivity.

**Table 3:** Results of orthogonal test scheme.

	A/Pa	B/min	C/W	D	(y_i_)/V
1	1	1	1	1	5.965
2	1	2	2	2	4.502
3	1	3	3	3	3.231
4	2	1	2	3	11.612
5	2	2	3	1	6.783
6	2	3	1	2	7.271
7	3	1	3	2	7.481
8	3	2	1	3	7.503
9	3	3	2	1	5.001
$K_{j1}$	13.698	25.058	20.739	17.749	K = 59.349 *P* = 391.367 *Q* = 437.274
$K_{j2}$	25.666	18.788	21.115	19.254	
$K_{j3}$	19.985	15.503	17.495	22.346	
$Q_{j}$	415.260	407.078	394.008	395.029	
$S_{j}^{2}$	23.893	15.711	2.641	3.662	$S_{T}^{2}$ = 45.907

It is evident that:

*K*_11_ < *K*_13_ < *K*_12_

*K*_23_ < *K*_22_ < *K*_21_

*K*_33_ < *K*_31_ < *K*_32_

Based on the analysis of the sum of squared dispersions, it is evident that the optimal combination is A_1_B_3_C_3_. Consequently, it can be inferred that when utilizing the average voltage drop of a copper–tin alloy film as the evaluation criterion, the combination exhibiting the highest conductivity is A_1_B_3_C_3_, which corresponds to the optimal process parameters of a working pressure of 0.065 Pa, a sputtering time of 20 min, and a sputtering power of 70 W.

### 3.2. Influence of Process Parameters on the Uniformity of Copper–Tin Alloy Film

**Figure 3:**
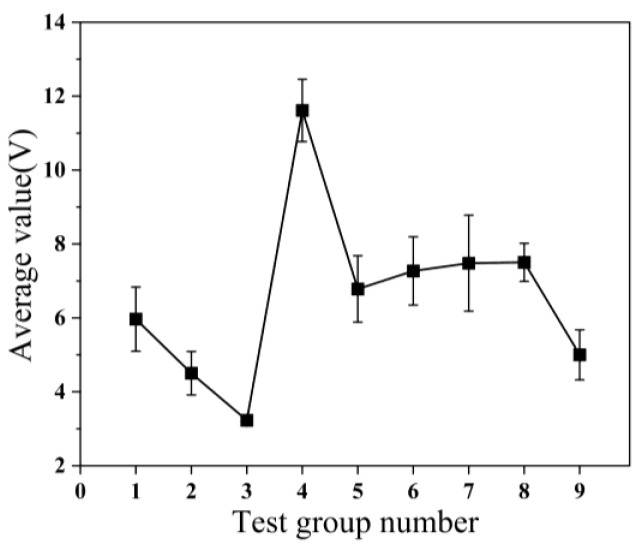
Standard deviation diagram.

**Figure 4:**
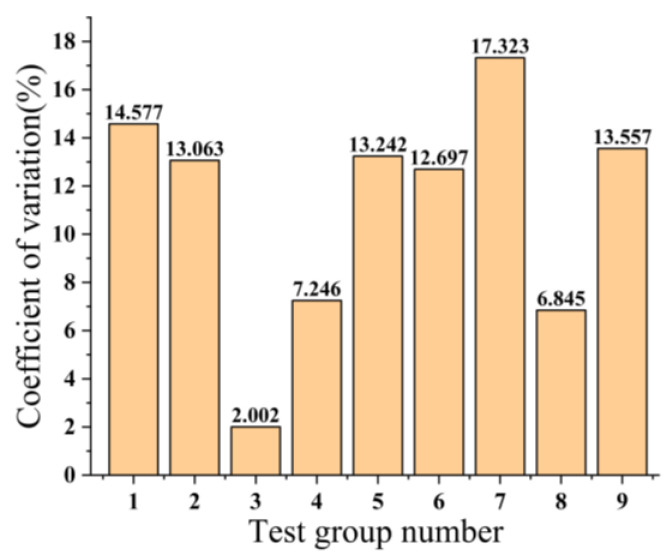
Coefficient of variation for 9 groups of experiments.

Figure 3 shows the standard deviation of the nine sets of tests. The standard deviation is a statistic that measures the dispersion of data by quantifying the average deviation between each data point and the mean. Figure 4 displays the coefficient of variation (CV) observed in the nine trials. The CV, which quantifies the standard deviation of the measured sample as a percentage of the sample mean, serves as a measure of the dispersion of the sample data. A smaller CV indicates a lower level of dispersion in the sample data, thereby implying a higher degree of uniformity. The uniformity of the copper–tin alloy film is also an essential criterion for assessing its quality. The calculation formula for the CV is as follows:

Coefficient of variation = sample standard deviation/sample mean × 100%

(9) $$Sample\;standard\;deviation=\sqrt{\frac{\sum_{i=1}^{n}{\left(x_{i}-\bar{x}\right)}^{2}}{n-1}}$$

Orthogonal tests can compare the effect of the level of another factor (A) on the test of changes in two factors (B, C). Because the level changes of the two factors (B, C) are regular and evenly dispersed, the change in the test index is mainly caused by the difference in the individual level of the other factor (A). Table 4 presents the division of the nine combinations of the orthogonal test into three groups, wherein only A_1_, A_2_, and A_3_ partake in the test, while the remaining factors B and C participate an equal number of times across the three groups. Within the three groups of trials, the remaining factors exhibit an equal contribution to the overall sum of coefficients of variation, thus suggesting a random interference. This comparison disregards the impact of additional variables, solely represents the influence of altering the level of factor A on the cumulative coefficients of variation, and can be employed to examine the consequences of modifying the value of various levels of factor A on the cumulative coefficients of variation. The same principle applies when scrutinizing other factors.

**Figure 5:**
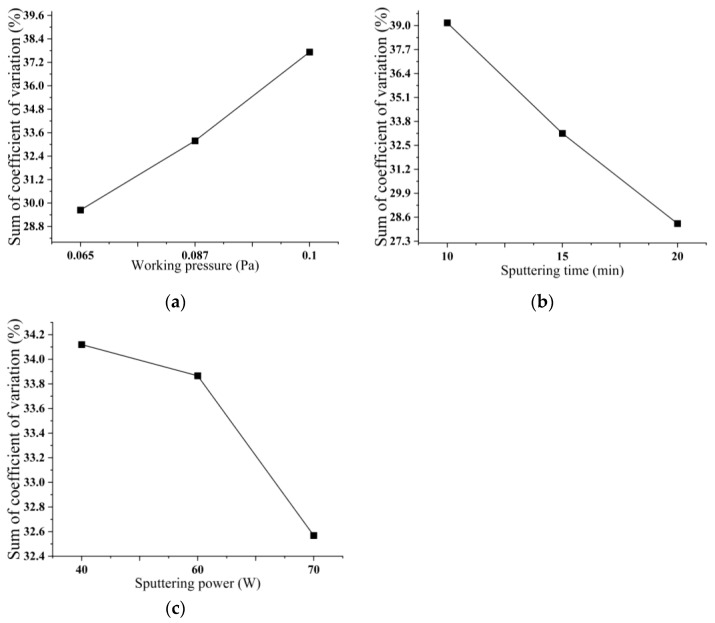
Relationship between different factors and the sum of coefficient of variation. (**a**) The relationship between working pressure and the sum of coefficient of variation; (**b**) The relationship between sputtering time and the sum of coefficient of variation; (**c**) The relationship between sputtering power and the sum of coefficient of variation.

As can be seen from Figure 5, as the working pressure increases, the sum of the coefficients of variation gradually increases, and the uniformity of the copper–tin alloy film deteriorates. Conversely, an increase in sputtering time and sputtering power results in a decrease in the sum of the coefficients of variation, suggesting an improvement in the uniformity of the film. Notably, the working pressure and sputtering time exert a more significant impact on the uniformity of the copper–tin alloy film, while the influence of sputtering power is minimal.

### 3.3. Influence of Process Parameters on Deposition Rate

**Figure 6:**
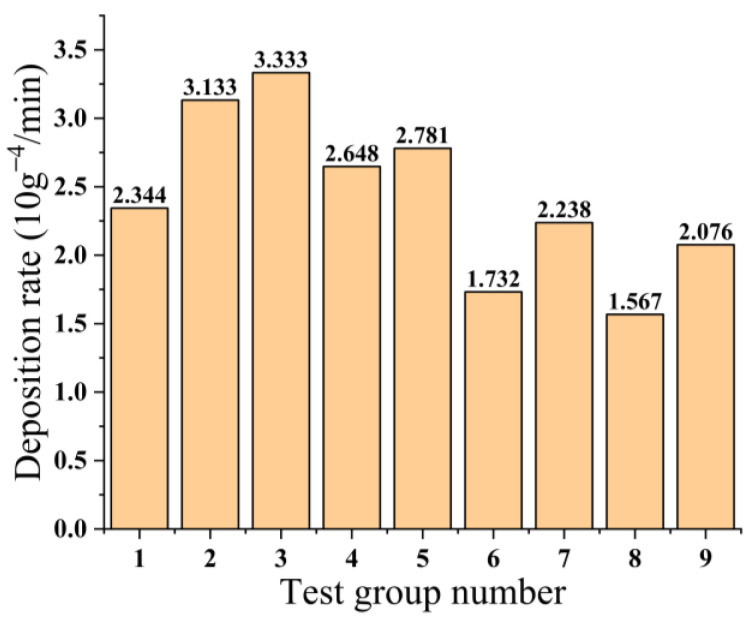
Deposition rate of 9 sets of experiments.

**Figure 7:**
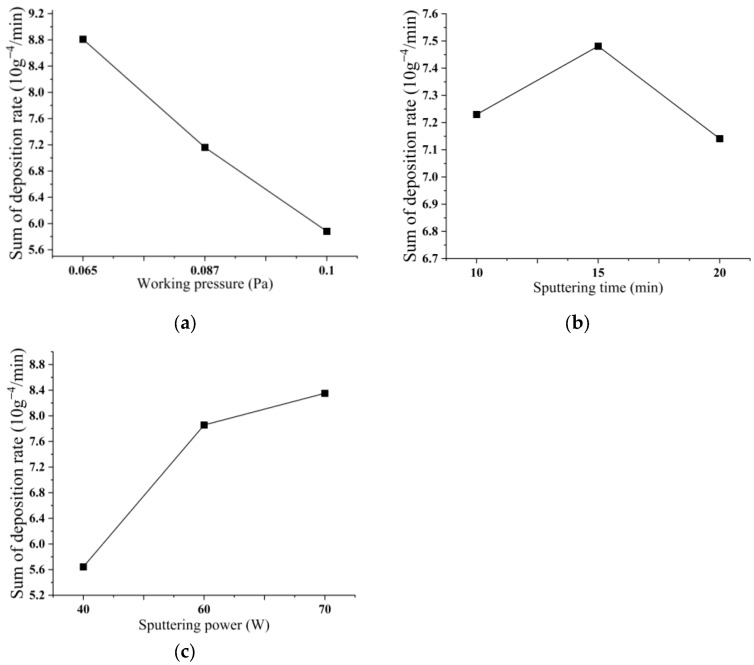
Relationship between different factors and the sum of deposition rate. (**a**) The relationship between working pressure and the sum of deposition rate; (**b**) The relationship between sputtering time and the sum of deposition rate; (**c**) The relationship between sputtering power and the sum of deposition rate.

Figure 6 illustrates the deposition rate of nine test groups, which serves as an indicator for the velocity at which the copper–tin alloy thin film is deposited. Figure 7 reveals that various factors exert distinct influences on the cumulative deposition rates. As the level of working pressure intensifies, the cumulative deposition rates correspondingly diminish. As the duration of sputtering time extends, the cumulative deposition rates exhibit a pattern of initial augmentation followed by subsequent decline. At a sputtering time of 15 min, the cumulative deposition rates reach their zenith. Conversely, the cumulative deposition rates escalate with an increase in sputtering power. Among the factors examined, the working pressure exerts the most pronounced influence on the deposition rate, while the impact of sputtering time is comparatively minimal.

### 3.4. Influence of Process Parameters on Target Current and Operating Voltage

**Figure 8:**
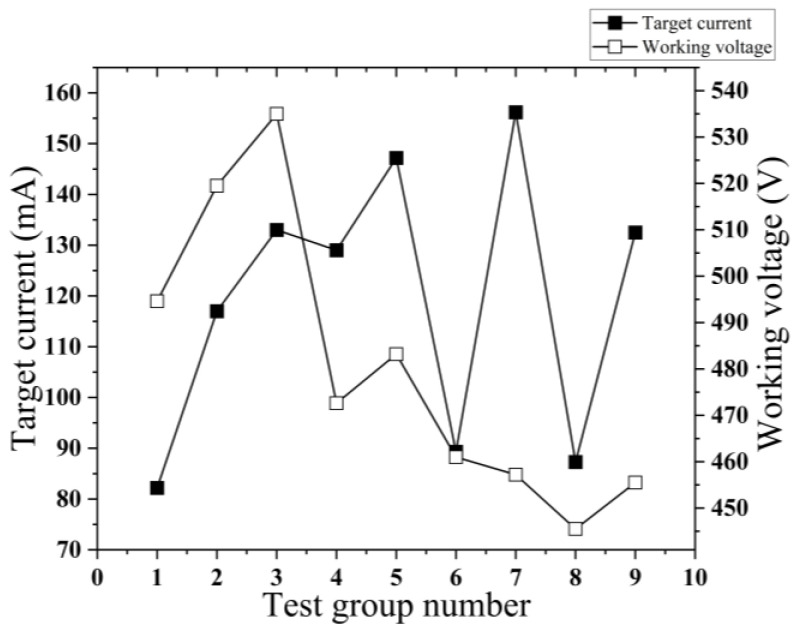
Target current and working voltage of 9 groups of tests.

**Figure 9:**
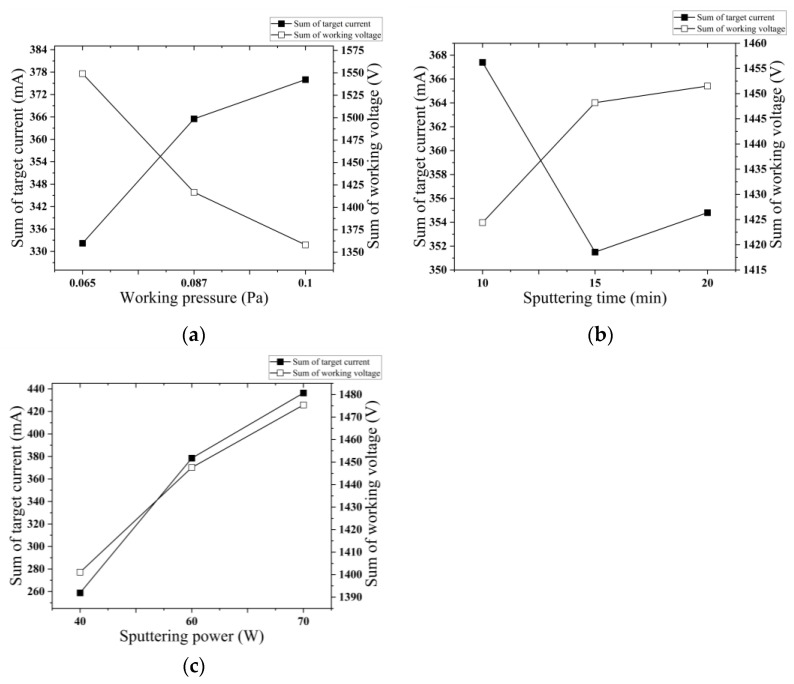
Relationship between different factors and the sum of target current and working voltage. (**a**) The relationship between working pressure and the sum of target current and working voltage; (**b**) The relationship between sputtering time and the sum of target current and working voltage; (**c**) The relationship between sputtering power and the sum of target current and working voltage.

Figure 8 depicts the diagram illustrating the target current and operating voltage for the nine sets of tests. As can be seen from Figure 9, the sum of the target currents increases with the increase in the operating voltage, and the sum of the operating voltages decreases with the increase in the operating voltage. As the sputtering time increases, the sum of the target currents decreases first and then increases, and the sum of the target currents is the smallest at 15 min. The operating voltage exhibits an increase as the sputtering time progresses. The correlation between sputtering power and target current and operating voltage demonstrates a nearly identical trend, whereby an increase in sputtering power leads to a simultaneous increase in both variables. The influence of sputtering power on the target current is the most significant, while the impact of sputtering time on the target current is the least pronounced. The influence of working pressure on the working voltage is found to be the most significant, while the impact of sputtering time on the working voltage is observed to be the least significant.

### 3.5. Microscopic Analysis of Samples

Based on the aforementioned orthogonal test results, it is evident that the magnetron sputtering process parameters yielding the highest conductivity are a working pressure of 0.065 Pa, sputtering power of 70 W, and sputtering time of 20 min. The outcomes of FT-IR detection, XRD detection, and SEM detection are presented below.

**Figure 10:**
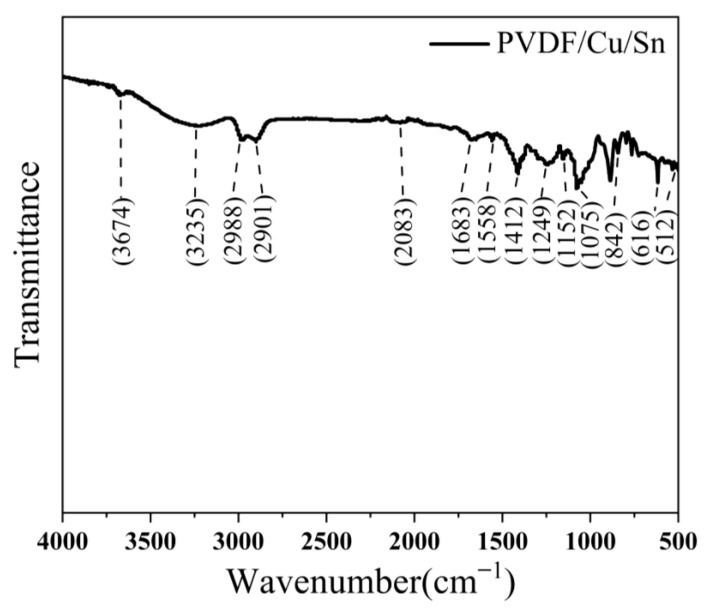
FT-IR detection results.

**Figure 11:**
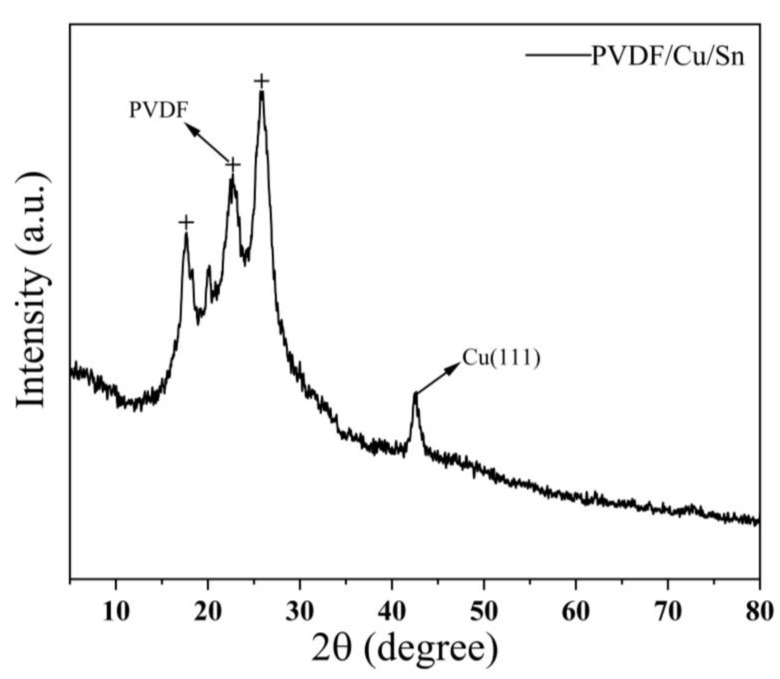
XRD detection results.

Figure 10 depicts the infrared spectroscopic analysis of the sample prepared using the optimal process parameters. The peak observed at around 512 cm^−1^ corresponds to the deformed vibration of both the C-F bond and the C-H bond, which is a characteristic bond in PVDF. Additionally, the peak observed at approximately 616 cm^−1^ signifies a combination of the C-CF_2_ bond and CF_2_ bond vibrations, which are also recognized as typical peaks of PVDF. At approximately 842 cm^−1^, the observed peak signifies the vibrational expansion of the CF_2_ bond resulting from the interaction between copper or tin and fluorine atoms within PVDF molecules, leading to the formation of chemical bonds. At around 1075 cm^−1^, the peak potentially corresponds to the vibration of C-F bonds, although it could also indicate a minor presence of OH bonds. At approximately 1152 cm^−1^, the observed peak can be attributed to the deformation vibration of a combination of C-F and C-H bonds. Similarly, around 1249 cm^−1^, the peak is likely indicative of the deformed vibration of a mixture of C-F and CF_2_ bonds. Furthermore, at approximately 1412 cm^−1^, the observed peak may signify a telescopic vibration of a combination of C-F and CF^2^ bonds potentially influenced by certain chemical bonds present in copper or tin. At approximately 1558 cm^−1^, the observed peak corresponds to the telescopic vibration of the C=C bond, which is a prevalent characteristic of unsaturated bonds in polyvinylidene fluoride (PVDF). Similarly, at around 1683 cm^−1^, the peak typically signifies the telescopic vibration of the C=O bond. Furthermore, at approximately 2083 cm^−1^, the peak potentially signifies vibrations arising from specific chemical bonds formed between PVDF and copper or tin. The peak observed at approximately 2901 cm^−1^ and 2988 cm^−1^ corresponds to a telescopic vibration associated with the C-H bond. At around 3235 cm^−1^, the peak could potentially arise from a limited number of OH functional groups or be attributed to the telescopic vibration of C-H. Conversely, the peak observed at 3674 cm^−1^ may indicate the presence of gaseous residue or an interference signal, as this particular position is not typically associated with common bonds found in PVDF. Figure 11 presents the XRD analysis diagram of the sample prepared using the optimal process parameters. The crystal structure of the PVDF polymer is characterized by a relatively intricate nature, with its chain segments having the ability to adopt at least four distinct crystal forms. Generally, the predominant crystalline form of PVDF is the α crystal form, which corresponds to the TGTG conformation. The graph illustrates that the diffraction peaks observed within the range of 18° to 28° are indicative of the characteristic diffraction peaks associated with PVDF. Additionally, the diffraction peak located at approximately 42.75° corresponds to the diffraction peak attributed to Cu.

**Figure 12:**
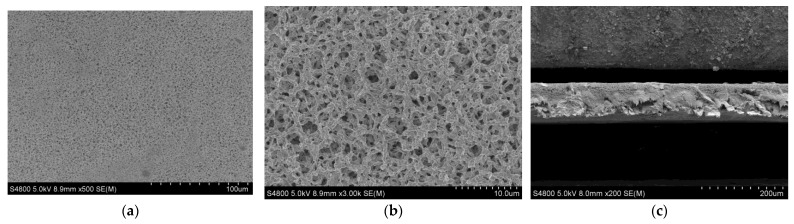
SEM detection results. (**a**) Surface 500× detection chart; (**b**) Surface 3000× detection chart; (**c**) Cross-sectional 200× detection chart.

Based on the observations depicted in Figure 12a, it is evident that the metal film produced using the specified process parameter exhibits a uniform and flat surface devoid of noticeable defects. In Figure 12b, the magnetron sputtering experiment reveals a layer-by-layer deposition of the metal film in an overlapping manner, which is characteristic of magnetron sputtering. Consequently, voids are present on the surface of the resulting metal film, although a continuous metal film is still present beneath these voids. Figure 12c demonstrates the formation of a Cu-Tn alloy film on both sides of the PVDF film with the metal film being comparatively thinner than the PVDF film.

## 4. Conclusions

In order to prepare a metal film with good electrical conductivity and uniformity, an orthogonal test is designed. The influence of different factors and levels on the conductivity of metal film was studied, and the process parameters were optimized. Tests show that the coefficient of variation and deposition rate, target current, and operating voltage of copper–tin alloy film is affected by the working voltage, sputtering time, and sputtering power.

When evaluating the average voltage drop of a copper–tin alloy film as the criterion, the findings from nine test groups and the analysis of the sum of squares of the difference indicated that the copper–tin alloy film produced under the A_1_B_3_C_3_ condition exhibited the highest conductivity. The optimal process parameters were determined to be a working pressure of 0.065 Pa, a sputtering time of 20 min, and a sputtering power of 70 W. The influence on the electrical conductivity of Cu-Tn alloy thin films can be attributed to the working pressure, sputtering time, and sputtering power, accounting for 52.047%, 34.224%, and 5.753%, respectively. Collectively, these factors contribute to 92.024% of the overall effect. It was observed that variations in the operating pressure, sputtering time, and sputtering power all had an impact on the coefficient of variation and deposition rate as well as the target current and operating voltage of the copper–tin alloy film. The uniformity of copper–tin alloy film is primarily influenced by sputtering time, with sputtering power having a lesser impact. Deposition velocity is primarily affected by working pressure, with sputtering time having a minimal effect. The target current is most significantly influenced by sputtering power, while sputtering time has a minimal impact. The operating voltage is most affected by working pressure and least affected by sputtering time.

## Figures and Tables

**Table 1:** Meter head design.

Factor	A	B	C	D
Number	1	2	3	4

**Table 2:** Test plan table.

	A/Pa	B/min	C/W
1	0.065 (1)	10 (1)	40 (1)
2	0.065	15 (2)	60 (2)
3	0.065	20 (3)	70 (3)
4	0.087 (2)	10	60
5	0.087	15	70
6	0.087	20	40
7	0.1 (3)	10	70
8	0.1	15	40
9	0.1	20	60

**Table 4:** Experimental combinations of factors A affecting the coefficient of variation.

Group 1		Group 2		Group 3	
	B_1_C_1_		B_1_C_2_		B_1_C_3_
A_1_	B_2_C_2_	A_2_	B_2_C_3_	A_3_	B_2_C_1_
	B_3_C_3_		B_3_C_1_		B_3_C_2_

## Data Availability

Not applicable.
